# Detecting Dysglycemia Using the 2015 United States Preventive Services Task Force Screening Criteria: A Cohort Analysis of Community Health Center Patients

**DOI:** 10.1371/journal.pmed.1002074

**Published:** 2016-07-12

**Authors:** Matthew J. O’Brien, Ji Young Lee, Mercedes R. Carnethon, Ronald T. Ackermann, Maria C. Vargas, Andrew Hamilton, Nivedita Mohanty, Sarah S. Rittner, Jessica N. Park, Amro Hassan, David R. Buchanan, Lei Liu, Joseph Feinglass

**Affiliations:** 1 Division of General Internal Medicine and Geriatrics, Northwestern University Feinberg School of Medicine, Chicago, Illinois, United States of America; 2 Center for Community Health, Institute for Public Health and Medicine, Northwestern University Feinberg School of Medicine, Chicago, Illinois, United States of America; 3 Department of Preventive Medicine, Northwestern University Feinberg School of Medicine, Chicago, Illinois, United States of America; 4 Alliance of Chicago Community Health Services, L3C, Chicago, Illinois, United States of America; 5 Erie Family Health Center, Chicago, Illinois, United States of America; 6 Department of Pediatrics, Northwestern University Feinberg School of Medicine, Chicago, Illinois, United States of America; 7 Robert H. Lurie Comprehensive Cancer Center, Northwestern University, Chicago, Illinois, United States of America; University of Cambridge, UNITED KINGDOM

## Abstract

**Background:**

In 2015, the United States Preventive Services Task Force (USPSTF) recommended targeted screening for prediabetes and diabetes (dysglycemia) in adults who are aged 40 to 70 y old and overweight or obese. Given increasing prevalence of dysglycemia at younger ages and lower body weight, particularly among racial/ethnic minorities, we sought to determine whether the current screening criteria may fail to identify some high-risk population subgroups.

**Methods and Findings:**

We investigated the performance of the 2015 USPSTF screening recommendation in detecting dysglycemia among US community health center patients. A retrospective analysis of electronic health record (EHR) data from 50,515 adult primary care patients was conducted. Longitudinal EHR data were collected in six health centers in the Midwest and Southwest. Patients with a first office visit between 2008 and 2010 were identified and followed for up to 3 y through 2013. We excluded patients who had dysglycemia at baseline and those with fewer than two office visits during the follow-up period. The exposure of interest was eligibility for screening according to the 2015 USPSTF criteria. The primary outcome was development of dysglycemia during follow-up, determined by: (1) laboratory results (fasting/2-h postload/random glucose ≥ 100/140/200 mg/dL [5.55/7.77/11.10 mmol/L] or hemoglobin A1C ≥ 5.7% [39 mmol/mol]); (2) diagnosis codes for prediabetes or type 2 diabetes; or (3) antidiabetic medication order. At baseline, 18,846 (37.3%) participants were aged ≥40 y, 33,537 (66.4%) were overweight or obese, and 39,061 (77.3%) were racial/ethnic minorities (34.6% Black, 33.9% Hispanic/Latino, and 8.7% Other). Overall, 29,946 (59.3%) patients had a glycemic test within 3 y of follow-up, and 8,478 of them developed dysglycemia. Only 12,679 (25.1%) patients were eligible for screening according to the 2015 USPSTF criteria, which demonstrated the following sensitivity and specificity (95% CI): 45.0% (43.9%–46.1%) and 71.9% (71.3%–72.5%), respectively. Racial/ethnic minorities were significantly less likely to be eligible for screening yet had higher odds of developing dysglycemia than whites (odds ratio [95% CI]: Blacks 1.24 [1.09–1.40]; Hispanics 1.46 [1.30–1.64]; and Other 1.33 [1.16–1.54]). In addition, the screening criteria had lower sensitivity in all racial/ethnic minority groups compared to whites. Limitations of this study include the ascertainment of dysglycemia only among patients with available test results and findings that may not be generalizable at the population level.

**Conclusions:**

Targeted diabetes screening based on new USPSTF criteria may detect approximately half of adult community health center patients with undiagnosed dysglycemia and proportionately fewer racial/ethnic minorities than whites. Future research is needed to estimate the performance of these screening criteria in population-based samples.

## Abstract 2

New diabetes screening criteria in the United States do not fully diagnose all patients with dysglycemia, Matthew O'Brien and colleagues report.

## Author Summary

**Why Was This Study Done?:**

- In October 2015, the United States Preventive Services Task Force (USPSTF) issued a new diabetes screening recommendation for adults who are aged 40–70 y and overweight or obese.
- These screening criteria may miss high-risk groups who develop prediabetes and diabetes (together called dysglycemia) at younger ages or at a normal weight.

**What Did the Researchers Do and Find?:**

- We conducted a study of electronic health record data from adult primary care patients at US community health centers between 2008 through 2013.
- The goal of the study was to determine patient characteristics associated with being eligible for diabetes screening, receiving a screening test, and developing dysglycemia over a 3-y period.
- The USPSTF screening criteria identified approximately half of patients who developed dysglycemia and proportionately fewer cases of dysglycemia among racial/ethnic minorities than whites.

**What Do These Findings Mean?:**

- Primary care providers may miss a substantial proportion of patients with dysglycemia by following the current USPSTF screening recommendation.
- Providers may consider screening patients from racial/ethnic minority groups before the age and weight ranges recommended by the USPSTF.
- However, patients may incur out-of-pocket costs when their doctors obtain a diabetes screening test that does not follow the USPSTF recommendation.

## Introduction

Diabetes affects over 410 million adults worldwide, with a growing global burden that is projected to increase [1,2]. In the US, 14% of adults have diabetes [3], which results in significant associated morbidity, mortality, and health care expenditures [4]. An estimated 38% of US adults have prediabetes [3], which comprises impaired glucose tolerance and impaired fasting glucose. These two high-risk states are characterized by elevations of plasma glucose above the normal range but below the diagnostic threshold for diabetes. Large randomized trials on three continents have demonstrated that structured, intensive lifestyle interventions can prevent or delay the onset of diabetes in adults with impaired glucose tolerance [5–8]. Of the many medication trials to prevent diabetes among those at high risk, metformin has been most widely studied and has found to be safe and effective at achieving this outcome [5,9,10]. The standards of care for treating diagnosed diabetes have been informed by an even larger evidence base [11]. The availability of effective treatments to prevent and treat diabetes underscores the importance of population-based strategies for identifying those at high risk.

Screening for prediabetes and diabetes (collectively referred to here as dysglycemia) has been recommended by many expert groups to improve evidence-based diabetes prevention and treatment efforts. In the US, the US Preventive Services Task Force (USPSTF) is an independent group of experts that reviews the evidence supporting screening tests and other clinical preventive services, and develops recommendations for their use in practice [12]. Federal law in the US requires that health insurance plans cover services recommended by the USPSTF without cost sharing [13]. In October 2015, this group issued a new recommendation to screen asymptomatic adults who are 40 to 70 y old and overweight or obese for dysglycemia using one of the following tests: hemoglobin A1C (A1C), fasting plasma glucose, or 2-h postload glucose during a 75-g oral glucose tolerance test [14].

Previous studies have investigated the effectiveness of various diabetes screening guidelines, but to our knowledge, none has yet examined the 2015 USPSTF criteria. Detecting dysglycemia is an increasingly important public health imperative, given rising rates of diabetes across all population subgroups. Recent research demonstrating a higher prevalence of diabetes at younger ages and lower body weight [15], particularly among racial/ethnic minorities [16–18], suggests that the USPSTF criteria may identify proportionately fewer cases of dysglycemia among certain high-risk groups. The objective of our study was to evaluate the performance of the 2015 USPSTF screening criteria among US community health center patients. We hypothesized that racial/ethnic minorities would be less likely to be detected by these screening criteria yet more likely to develop dysglycemia over time.

## Methods

### Ethics Statement

The research protocol was approved by each of the participating community health centers’ research evaluation boards and was deemed exempt from review by the Northwestern University Institutional Review Board.

### Data Source and Participants

In this retrospective cohort study, we analyzed longitudinal electronic health record (EHR) data from a US network of publicly funded community health centers between 2008 and 2013. This study is reported as per STROBE guidelines (S1 STROBE Checklist). EHR data from routine primary care encounters were collected retrospectively by Alliance of Chicago Community Health Services (Alliance), a Health Center Controlled Network and member of the Community Health Applied Research Network (CHARN). The affiliated clinics offer comprehensive clinical services to vulnerable populations including large proportions of women and racial/ethnic minorities [19]. Six community health centers in the CHARN network serving patients in the American Midwest and Southwest participated in this study [20]. We used structured query language to extract from a centralized data warehouse the following EHR data on patients receiving primary care in these centers: age, sex, self-reported race/ethnicity, insurance type, laboratory testing results, physical measurements (height, weight, and blood pressure), medications ordered, comorbidities (coded according to the International Classification of Diseases, Ninth Revision [ICD-9]) [21], and the date on which these data elements were collected.

Adult patients who were at least 18 y old were identified in the EHR between January 2008 and December 2010 based on their first office visit during this time period, defined as the index visit. Patients who had fewer than two subsequent office visits were excluded after examining all face-to-face encounters with a licensed primary care provider. We also excluded those with dysglycemia at baseline, determined by the following EHR criteria documented any time before the end of the calendar year in which patients attended the index visit: (1) ICD-9 diagnosis code for prediabetes or diabetes; (2) prescription order for any antidiabetic medication; and/or (3) glycemic testing result consistent with dysglycemia (fasting glucose ≥ 100 mg/dL [5.55 mmol/L], 2-h postload glucose ≥140 mg/dL [7.77 mmol/L], random glucose ≥ 200 mg/dL [11.10 mmol/L], or A1C ≥ 5.7% [39 mmol/mol]). These patients were excluded because the study’s primary outcome was incident dysglycemia during a 3-y follow-up period. In addition, we excluded five subjects with unknown sex. The final study population included 50,515 patients, who were followed after the index visit for up to 3 y through December 2013. We chose this duration of follow-up because it is the recommended time interval for dysglycemia screening [14]. Each patient was observed for a single follow-up period and was analyzed only once.

### Measures and Definitions

#### Screening eligibility, diabetes risk factors, and other covariates

According to the 2015 USPSTF criteria, we considered all patients who were aged 40 to 70 y old and overweight or obese to be eligible for screening. In addition to age and body mass index (BMI), we analyzed baseline data on each of the following diabetes risk factors as defined according to the American Diabetes Association (ADA): nonwhite race/ethnicity, hypertension, dyslipidemia, polycystic ovary syndrome, history of gestational diabetes, and family history of diabetes [22]. The ADA includes physical inactivity as a diabetes risk factor, which was not routinely collected in the EHR data source and therefore was not examined in this study. Race/ethnicity data were derived from self-reports obtained upon patient registration in the participating community health centers and used to create mutually exclusive categories. Nonwhite race and ethnicity categories included in our analysis were Black, Hispanic/Latino, and Other. All patients reporting Hispanic/Latino ethnicity were categorized as Hispanic/Latino in the analysis. The Other category comprised American Indians, Asians, Native Hawaiians or Other Pacific Islanders, biracial or multiracial individuals, and those with missing data for race/ethnicity. Definitions for each risk factor were based on a combination of EHR data elements: ICD-9 diagnosis codes, laboratory results, physical measurements, and medications prescribed (definitions presented in Table 1). We summed the number of diabetes risk factors, including age ≥ 45 y [22] and those mentioned above, to create an individual risk score. Sociodemographic variables including sex and insurance status were also assessed at the index visit and examined as covariates [23,24].

**Table 1:** Determination of diabetes, prediabetes, and other diabetes risk factors using electronic health record data^a^.

Condition or Risk Factor	ICD-9 Codes^b^	Laboratory Values or Clinical Measurements	Medication Prescriptions	Demographic Characteristics
Diabetes	250.00–250.93^c^	Hemoglobin A1C ≥6.5%; glucose: fasting (≥126 mg/dL), random (≥200 mg/dL), and 2-h (≥200 mg/dL)	Antidiabetic medication	N/A
Prediabetes	790.21, 790.22, 790.29 in absence of 250.00–250.93	Hemoglobin A1C 5.7%–6.4%; glucose: fasting (100–125 mg/dL) and 2-h (140–199 mg/dL)	No antidiabetic medication	N/A
Hypertension	401.0–401.9, 402.00–402.91, 404.00–404.93	Blood pressure >140/90 mmHg	Antihypertensive medication	N/A
Dyslipidemia	272.0–272.4	HDL cholesterol <35 mg/dL, triglycerides >250 mg/dL	Lipid-lowering medication	N/A
Overweight/Obesity^d^	278.00–278.02, V85.2–85.4	Body mass index ≥25 kg/m^2^	N/A	N/A
History of Gestational Diabetes	648.80–648.83, V12.21	N/A	N/A	N/A
History of Polycystic Ovary Syndrome	256.4	N/A	N/A	N/A
Family History of Diabetes	V18.0	N/A	N/A	N/A
Age	N/A	N/A	N/A	≥45 y old
Race/Ethnicity (Self-Reported)	N/A	N/A	N/A	White, Black, Hispanic, Other

HDL, high-density lipoprotein; N/A, not applicable.

^a^ In order to be classified as having any of the listed conditions or risk factors, patients needed evidence of at least one of the criteria presented.

^b^ Diagnostic codes are based on the International Classification of Diseases, Ninth Revision (ICD-9).

^c^ ICD-9 codes for both type 1 and type 2 diabetes were used to exclude participants with dysglycemia at baseline. ICD-9 codes for type 2 diabetes were used to determine the development of dysglycemia during the follow-up period.

^d^ Body mass index (BMI) was calculated using measured height and weight to determine overweight/obesity status, defined as BMI ≥25 kg/m2. ICD-9 codes for overweight or obesity were used instead when measured BMI was an implausible value (defined as ≤15 or ≥75 kg/m2).

#### Receipt of diabetes screening and incident dysglycemia

Glycemic testing conducted during routine clinical encounters is often called opportunistic diabetes screening. This approach has proven effective for identifying undiagnosed dysglycemia in US practice-based settings and has also been advocated as a case finding strategy in low- and middle-income countries [25,26]. Following the current USPSTF guideline [14], we considered receiving at least one of the following glycemic tests during follow-up as screening for dysglycemia: fasting glucose, 2-h postload glucose, and A1C. According to the ADA [22], we also considered the results of random glucose tests, which may be more effective at detecting dysglycemia than fasting glucose tests, as receipt of opportunistic screening [27].

The development of dysglycemia was the primary outcome, ascertained using a combination of screening laboratory results, ICD-9 diagnosis codes, and prescribed medications. Patients with one of the following glycemic testing results were considered to have dysglycemia: fasting glucose ≥ 100 mg/dL (5.55 mmol/L), 2-h postload glucose ≥ 140 mg/dL (7.77 mmol/L), or A1C ≥ 5.7% (39 mmol/mol). Patients with random glucose ≥ 200 mg/dL (11.10 mmol/L) were classified as having diabetes and therefore included among those who developed dysglycemia [22]. A label of “fasting,” “random,” or “2-h postload” for all glucose results in the EHR enabled laboratory definitions of the outcome, which was based on the first result observed during the follow-up period. Establishing a diagnosis of diabetes or prediabetes requires confirmation by repeat testing, which was not assessed here. Dysglycemia was also defined by documentation of diagnosis codes for prediabetes or type 2 diabetes during follow-up. Patients with a new antidiabetic medication prescription were also considered to have dysglycemia. We classified patients who met any of the criteria described above as screening positive for dysglycemia.

### Statistical Analysis

We used summary statistics to characterize the entire study cohort with respect to all covariates at baseline. Chi-square tests were used to examine the association between diabetes screening eligibility according to 2015 USPSTF criteria (eligible versus ineligible) and the following indicators: (1) baseline characteristics, (2) receipt of screening during follow-up, and (3) development of clinically detected dysglycemia. Using data on USPSTF eligibility and dysglycemia incidence among patients who were screened during follow-up (*n* = 29,946), we assessed the performance characteristics of the USPSTF criteria (sensitivity, specificity, positive predictive value [PPV], and negative predictive value [NPV]) based on available screening test results. Estimation of these performance characteristics did not include those who were not screened during follow-up, in whom the incidence of dysglycemia was unknown. We conducted stratified analyses by race/ethnicity to examine the development of dysglycemia separately in these groups. Also in the screened population, we estimated the odds of developing clinically detected dysglycemia during follow-up using logistic regression adjusted for all the covariates, in addition to community health center site. Participants with missing race/ethnicity data were included in bivariate and multivariable analyses as part of the Other category. Those with missing data for insurance status were included in all analyses under a separate Missing category. In a sensitivity analysis, we estimated the performance characteristics of the USPSTF criteria including patients who also had undiagnosed dysglycemia at baseline, who would also be eligible for screening according to the recommendation. A *p*-value of <0.05 was considered significant for all statistical testing. All analyses were conducted using SAS, version 9.4 (SAS Institute, Cary, North Carolina).

Our team designed the analysis plan for this study before receiving the data and made no major departures from it. Table 1, presenting the criteria used to define our outcome and other clinical covariates, was used by our co-authors at Alliance to extract the EHR data used in our study. The age and insurance categories analyzed here represent the only departures from our initial analysis plan. The analysis was originally planned using the following categories for age and insurance status: 18–39, 40–70, and ≥71 y and Medicaid, Medicare, Private, Uninsured, and Other, respectively.

## Results

### Cohort Characteristics

**Fig 1:**
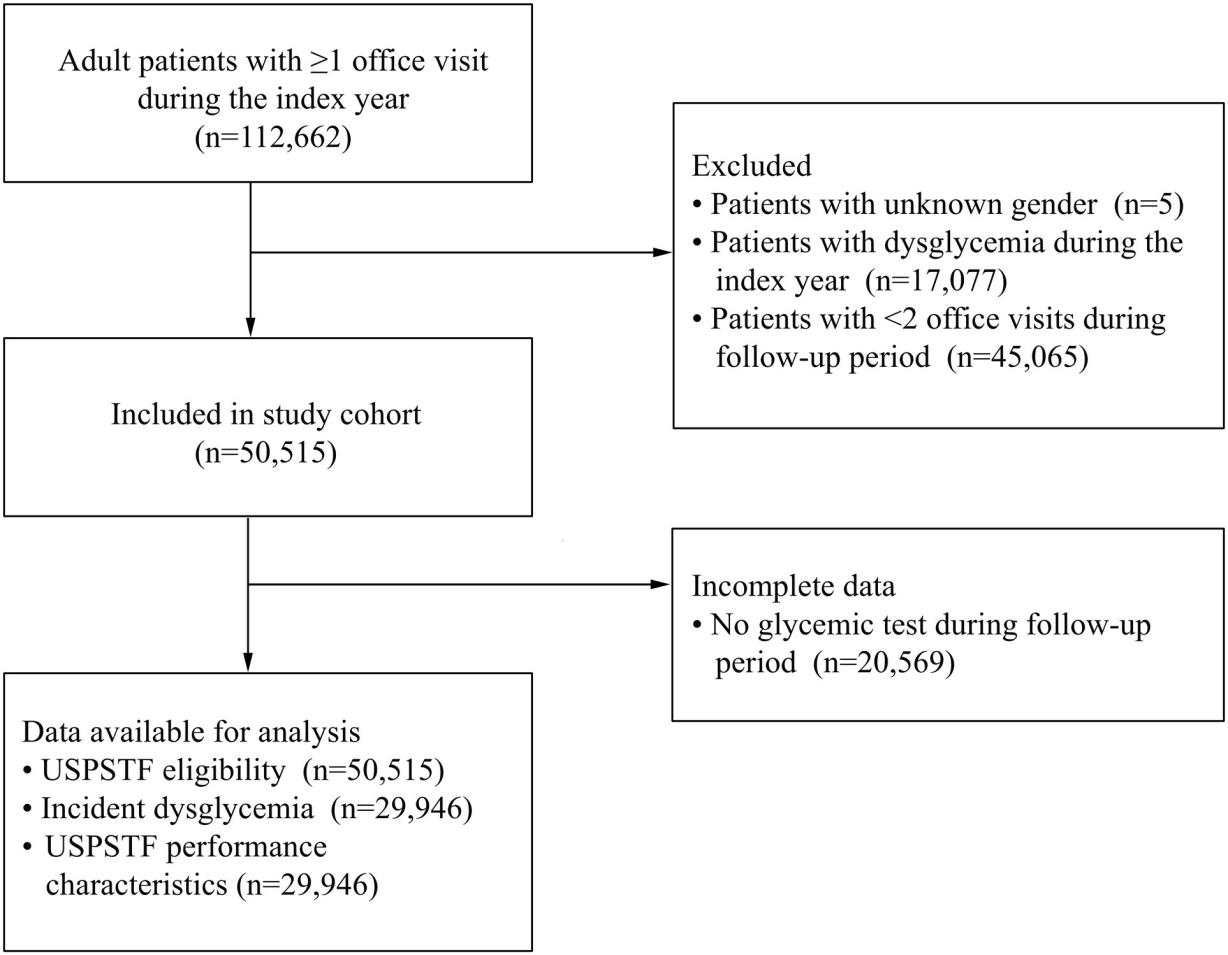
Flow diagram.

The Fig 1 flow diagram displays the numbers of the total patient population (*n* = 112,662), the patients excluded and reasons for their exclusion (*n* = 62,147), the participants comprising the study sample (*n* = 50,515), and those analyzed (*n* = 50,515). The mean follow-up time was 1.9 y and the maximum was 3.0 y. At baseline, the patient cohort was predominantly less than 40 y old (62.7%), overweight/obese (66.4%), nonwhite (77.3%), women (72.5%), and uninsured or publicly insured (74.3%). There were missing data for race/ethnicity (*n* = 977, 1.9%) and insurance status (*n* = 4,103, 8.1%), but not for other variables. Over 95% of the sample had at least one risk factor for diabetes, and 25.1% met current USPSTF criteria for diabetes screening. According to these criteria, all patients less than 40 y old or with a normal body weight would have been ineligible for screening. In addition, the following patient characteristics were significantly associated with being ineligible: nonwhite race/ethnicity, female sex, history of gestational diabetes, and polycystic ovary syndrome (Table 2).

**Table 2:** Baseline characteristics of adult community health center patients without dysglycemia by USPSTF screening eligibility.

Characteristics^a^	Total Patient Sample *n* (%)	Eligible *n* (%)	Ineligible *n* (%)
Number of Patients	50,515 (100)	12,657 (25.1)	37,858 (74.9)
Age, y			
18–39	31,669 (62.7)	0 (0.0)	31,669 (100)
40–59	15,066 (29.8)	10,719 (71.2)	4,347 (28.9)
≥60	3,780 (7.5)	1,938 (51.3)	1,842 (48.7)
Race/Ethnicity			
White	11,454 (22.7)	3,547 (31.0)	7,907 (69.0)
Black	17,501 (34.7)	4,980 (28.5)	12,521 (71.5)
Hispanic/Latino	17,147 (33.9)	3,140 (18.3)	14,007 (81.7)
Other^b^	4,413 (8.7)	990 (22.4)	3,423 (77.6)
Sex			
Male	13,919 (27.6)	4,821 (34.6)	9,098 (65.4)
Female	36,596 (72.5)	7,836 (21.4)	28,760 (78.6)
Insurance Status			
Medicaid	16,019 (31.7)	3,093 (19.3)	12,926 (80.7)
Medicare	4,353 (8.6)	2,192 (50.4)	2,161 (49.6)
Private	8,194 (16.2)	2,093 (25.5)	6,101 (74.5)
Uninsured	17,180 (34.0)	4,033 (23.5)	13,147 (76.5)
Other	666 (1.3)	205 (30.8)	461 (69.2)
Missing	4,103 (8.1)	1,041 (25.4)	3,062 (74.6)
Weight			
Normal	16,978 (33.6)	0 (0.0)	16,978 (100)
Overweight	15,466 (30.6)	5,731 (37.1)	9,735 (62.9)
Obese	18,071 (35.8)	6,926 (38.3)	11,145 (61.7)
Hypertension	11,376 (22.5)	6,033 (53.0)	5,343 (47.0)
Dyslipidemia	6,868 (13.6)	3,799 (55.3)	3,069 (44.7)
Polycystic Ovary Syndrome	171 (0.3)	4 (2.3)	167 (97.7)
History of Gestational Diabetes	655 (1.3)	38 (5.8)	617 (94.2)
Family History of Diabetes	7,058 (14.0)	1,789 (25.4)	5,269 (74.7)
Number of Diabetes Risk Factors			
0	2,352 (4.7)	0 (0.0)	2,352 (100)
1	11,189 (22.2)	342 (3.1)	10,847 (96.9)
2	19,255 (38.1)	2,503 (13.0)	16,752 (87.0)
≥3	17,719 (35.1)	9,812 (55.4)	7,907 (44.6)

^a^ All participant characteristics were significantly associated with USPSTF eligibility at a significance level of *p* < 0.001 except family history of diabetes (*p* = 0.55).

^b^ This category comprised American Indians, Asians, Native Hawaiians and Other Pacific Islanders, biracial or multiracial individuals, and those with missing data for race/ethnicity (*n* = 977).

### Receipt of Screening and Development of Dysglycemia

Overall, 29,946 patients (59.3% of the total sample) underwent a screening test within 3 y of the index visit. Screening was completed in 77.8% of patients who were eligible according to the 2015 USPSTF criteria and 53.1% of those who were not. Based on available test results, a total of 8,478 patients developed dysglycemia during the follow-up period (5,960 with prediabetes [70.3% of dysglycemia cases] and 2,518 with diabetes). The following proportion of dysglycemia cases were identified by each glycemic test: A1C (77.9%), fasting glucose (18.2%), 2-h postload glucose (0.7%), and random glucose (3.1%). Only 45.0% of those who developed dysglycemia would have met current USPSTF criteria. Among the dysglycemia cases missed by the USPSTF criteria, 77.7% were less than 40 y old and 29.3% had a normal body weight. The yield of screening for detecting dysglycemia was 38.8% among those who were eligible and 23.2% among those who were not (Table 3). Using data from all patients who had a screening test during the follow-up period, the 2015 USPSTF criteria had the following test performance characteristics (95% CI) for identifying dysglycemia: sensitivity 45.0% (43.9%–46.1%), specificity 71.9% (71.3%–72.5%), PPV 38.8% (37.8%–39.7%), and NPV 76.8% (76.2%–77.4%). A sensitivity analysis including individuals who had undiagnosed dysglycemia at baseline did not substantively impact the reported USPSTF performance characteristics.

**Table 3:** Receipt of screening and development of clinically detected dysglycemia within 3 y by USPSTF eligibility.

Characteristics^a^	Received Screening Test^b^ (*n* = 29,946)		Developed Dysglycemia^c^ (*n* = 8,478)	
	Eligible *n* (%)	Ineligible *n* (%)	Eligible *n* (%)	Ineligible *n* (%)
Number of patients	9,844 (32.9)	20,102 (67.1)	3,815 (45.0)	4,663 (55.0)
Age, y				
18–39	0 (0.0)	15,781 (100)	0 (0)	3,624 (100)
40–59	8,208 (73.8)	2,916 (26.2)	3,116 (84.8)	557 (15.2)
≥60	1,636 (53.8)	1,405 (46.2)	699 (59.2)	482 (40.8)
Sex				
Male	3,846 (40.4)	5,680 (59.6)	1,338 (57.2)	1,003 (42.8)
Female	5,998 (29.4)	14,422 (70.6)	2,477 (40.4)	3,660 (59.6)
Insurance Status				
Medicaid	2,498 (26.6)	6,907 (73.4)	1,020 (39.8)	1,546 (60.2)
Medicare	1,897 (53.7)	1,638 (46.3)	798 (62.8)	472 (37.2)
Private	1,593 (32.7)	3,273 (67.3)	524 (50.1)	521 (49.9)
Uninsured	3,043 (29.9)	7,145 (70.1)	1,234 (39.9)	1,860 (60.1)
Other	144 (37.8)	237 (62.2)	43 (39.8)	65 (60.2)
Missing	669 (42.6)	902 (57.4)	196 (49.6)	199 (50.4)
Weight				
Normal	0 (0.0)	8,725 (100)	0 (0)	1,365 (100)
Overweight	4,337 (46.5)	4,980 (53.5)	1,357 (56.7)	1,037 (43.3)
Obese	5,507 (46.3)	6,397 (53.7)	2,458 (52.1)	2,261 (47.9)
Hypertension	4,955 (57.5)	3,661 (42.5)	1,975 (64.4)	1,093 (35.6)
Dyslipidemia	3,237 (58.0)	2,345 (42.0)	1,267 (65.5)	668 (34.5)
Polycystic Ovary Syndrome	4 (4.3)	90 (95.7)	3 (7.0)	40 (93.0)
History of Gestational Diabetes	25 (9.3)	244 (90.7)	13 (13.3)	85 (86.7)
Family History of Diabetes	1,439 (33.0)	2,916 (67.0)	589 (45.4)	708 (54.6)
Number of Diabetes Risk Factors				
0	0	998 (100)	0 (0)	71 (100)
1	220 (4.2)	5,071 (95.8)	37 (5.3)	662 (94.7)
2	1,763 (16.6)	8,885 (83.4)	567 (19.9)	2,280 (80.1)
≥3	7,861 (60.4)	5,148 (39.6)	3,211 (66.1)	1,650 (33.9)

^a^ All participant characteristics were significantly associated with USPSTF eligibility at a significance level of *p* < 0.001, both among all patients who received a screening test and those who developed dysglycemia.

^b^ Patients who had an available result for one of the following glycemic tests were considered to have received a screening test: fasting glucose, random glucose, 2-h postload glucose during a 75-g oral glucose tolerance test, and hemoglobin A1C.

^c^ The development of dysglycemia was ascertained among all patients who received a screening test (*n* = 29,946).

The multivariable model of incident dysglycemia adjusted for all potentially confounding variables, including community health center site. The following demographic and clinical characteristics were significantly associated with developing dysglycemia during follow-up: age ≥ 40 y, overweight and obesity, nonwhite race/ethnicity, hypertension, polycystic ovary syndrome, history of gestational diabetes, and family history of diabetes. The odds of developing dysglycemia increased with greater numbers of diabetes risk factors (Table 4).

**Table 4:** Odds of developing clinically detected dysglycemia within 3 y among patients who received screening (*n* = 29,946).

Characteristics	Odds Ratio (95% CI)^a^
Age, y	
18–39	REF
40–59	1.74 (1.62–1.88)
≥60	1.92 (1.70–2.16)
Race/Ethnicity	
White	REF
Black	1.24 (1.09–1.40)
Hispanic/Latino	1.46 (1.30–1.64)
Other^b^	1.33 (1.16–1.54)
Sex	
Male	REF
Female	0.99 (0.93–1.06)
Insurance Status	
Medicaid	REF
Medicare	1.17 (1.05–1.30)
Private	0.93 (0.85–1.02)
Uninsured	1.08 (1.01–1.16)
Other	0.89 (0.71–1.13)
Missing	0.70 (0.61–0.80)
Weight	
Normal	REF
Overweight	1.31 (1.19–1.44)
Obese	2.45 (2.23–2.69)
Hypertension	1.12 (1.04–1.21)
Dyslipidemia	1.00 (0.93–1.08)
Polycystic Ovary Syndrome	2.24 (1.45–3.45)
History of Gestational Diabetes	1.31 (1.01–1.71)
Family History of Diabetes	1.09 (1.01–1.17)
Number of Diabetes Risk Factors^c^	
0	REF
1	1.98 (1.54–2.56)
2	4.77 (3.73–6.09)
≥3	7.79 (6.10–9.94)

REF, reference group.

^a^ Odds ratios (95% confidence intervals [CIs]) are adjusted for age, race/ethnicity, sex, insurance status, weight, hypertension, dyslipidemia, polycystic ovary syndrome, history of gestational diabetes, family history of diabetes, and community health center site.

^b^ This category comprised American Indians, Asians, Native Hawaiians and Other Pacific Islanders, biracial or multiracial individuals, and those with missing data for race/ethnicity (*n* = 977).

^c^ The odds ratios for number of diabetes risk factors are unadjusted.

### Screening Eligibility, Receipt, and Development of Dysglycemia by Race/Ethnicity

**Fig 2:**
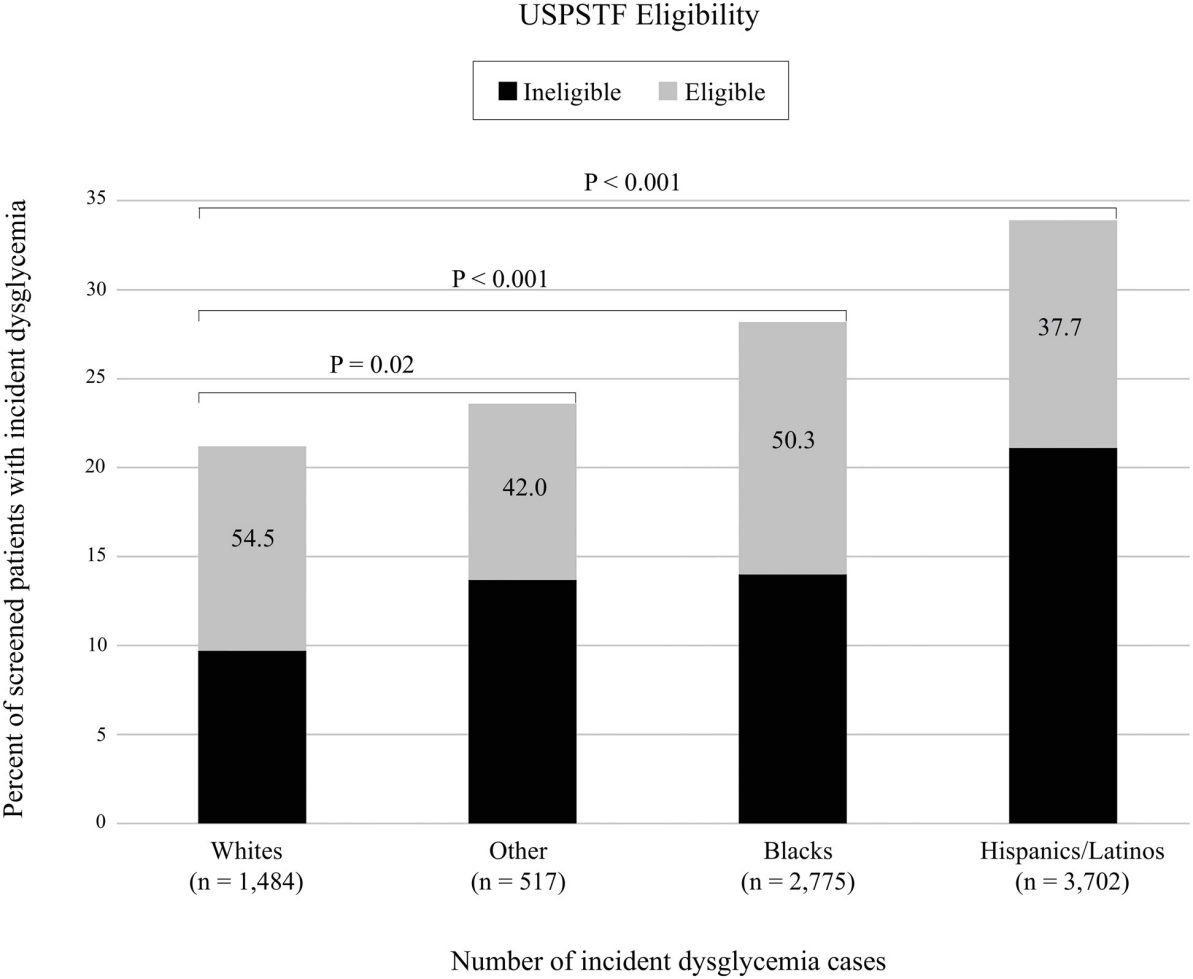
Development of clinically detected dysglycemia within 3 y among patients who received screening by race/ethnicity and USPSTF eligibility (*n* = 29,946). *P*-values were derived from pairwise comparisons of USPSTF eligibility among racial/ethnic groups using Chi-square tests. Numbers displayed within the bars represent the probability of being eligible for screening among those who developed dysglycemia (i.e., sensitivity). USPSTF, US Preventive Services Task Force.

Racial/ethnic minorities were significantly less likely to be eligible for screening based on the USPSTF recommendation than were whites, with the greatest disparity occurring among Hispanics/Latinos (Table 2). A higher proportion of racial/ethnic minorities who were screened developed clinically detected dysglycemia during the follow-up period (Fig 2). This was supported by multivariable logistic regression analysis showing higher odds of dysglycemia among all racial/ethnic minority groups compared to whites (odds ratio [95% CI] for Blacks 1.24 [95% CI, 1.09–1.40]; Hispanics 1.46 [95% CI, 1.30–1.64]; and Other 1.33 [95% CI, 1.16–1.54]) (Table 4). In addition, dysglycemia cases among minority patients were less likely to be identified by the USPSTF screening criteria (i.e., lower sensitivity) than were cases among whites. The sensitivity (95% CI) of the criteria was 54.5% (52.0%–57.1%) in whites, 50.3% (48.4%–52.1%) in Blacks, 42.0% (37.7%–46.2%) in Other, and 37.7% (36.1%–39.2%) in Hispanics/Latinos (Fig 2). The lower sensitivity observed among racial/ethnic minorities reflects the greater proportion of patients who developed dysglycemia at a normal weight and ages less than 40 within these groups, relative to whites (Table 5).

**Table 5:** Receipt of screening and development of clinically detected dysglycemia by race/ethnicity, age < 40 y, and normal weight status.

Race/Ethnicity	Total *n*	Received Screening *n* (%)^a^	Developed Dysglycemia *n* (%)^b^
White			
Age < 40 y old	5,283	2,543 (48.1)	333 (13.1)
Normal weight	4,945	2,658 (53.8)	359 (13.5)
Black			
Age < 40 y old	10,512	4,631 (44.1)	1,165 (25.2)
Normal weight	5,160	2,494 (48.3)	433 (17.4)
Hispanic/Latino			
Age < 40 y old	13,016	7,476 (57.4)	2,295 (30.7)
Normal weight	5,085	2,793 (54.9)	553 (19.8)
Other			
Age < 40 y old	2,858	1,131 (39.6)	226 (20.0)
Normal weight	1,788	780 (43.6)	133 (17.1)

^a^ The denominator for the reported percentages is the total number of patients within each stratum.

^b^ The denominator for the reported percentages is the number of patients within each stratum who received screening.

## Discussion

Using longitudinal data from US community health center patients, we report eligibility for diabetes screening according to the 2015 USPSTF criteria, as well as receipt of screening and positive test results within a 3-y follow-up period. Overall, one-quarter of patients were eligible for screening, and almost 60% received a screening test during follow-up. Less than half of the patients who developed dysglycemia would have been eligible for screening according to current USPSTF criteria. Our findings also suggest that certain population groups that are at high risk for developing dysglycemia are more likely to be missed when using these criteria, particularly racial/ethnic minorities.

To our knowledge, this is the first study to examine USPSTF diabetes screening criteria released in October 2015. Using US population-based data in which all participants received glycemic testing, previous analyses tested the detection of dysglycemia according to a prior 2008 USPSTF recommendation to screen adults with hypertension. One study reported that 28.2% of US adults aged 18 y or older had hypertension and were therefore eligible to be screened according to the 2008 recommendation [28]. Screening adults based on hypertension alone was estimated to identify 53% of individuals with diabetes and 31% of those with prediabetes [29]. Sensitivity and specificity for detecting dysglycemia by the previous screening criterion (blood pressure >135/80 mmHg) were 40.6%–44.4% and 74.8%–78.0%, respectively [30,31]. Despite studying different screening criteria in nationally representative populations, these analyses reported remarkably similar performance characteristics to those we report in a clinic-based sample. Alternate diabetes screening criteria based on a greater number of risk factors generally found higher sensitivity and lower specificity than the 2008 USPSTF recommendation [29,31,32].

Our study found comparable rates of opportunistic screening with previous reports from US primary care settings [32–34]. Studies analyzing US survey data have found a lower prevalence of diabetes screening [31,35]. The discrepancy between estimates derived from clinical cohorts and population-based studies likely reflects an increased probability of those receiving health care to be screened and recall bias from using self-reported data to ascertain this outcome. We found a higher prevalence of screening in patients who would have been eligible based on the current USPSTF criteria, compared to those who would not be eligible. Screening was also more common among patients with other diabetes risk factors, except for gestational diabetes and polycystic ovary syndrome. Because rare conditions are infrequently captured in EHR data [36], our screening estimates for these patients may be biased. Opportunistic screening in patients with hypertension and hyperlipidemia may reflect monitoring of electrolytes, renal function, and liver function among those taking medication.

While diabetes screening occurred in approximately half of the patients who would not be eligible according to the 2015 USPSTF recommendation, this practice may change after implementation of the new screening criteria. Previous studies have reported good adherence to other USPSTF screening recommendations [37–40], which suggests that dysglycemia screening may occur less frequently among ineligible patients in the future. The lack of mandated insurance coverage for screening among ineligible patients in the US may also result in lower screening rates than those observed here [13]. This may be especially true in safety-net settings like those we studied, where patients are less able to afford out-of-pocket medical expenses [41].

Our sample presents a unique opportunity to investigate demographic groups that may be missed by the 2015 USPSTF screening criteria, given the proportion of young and lean patients who developed dysglycemia. We found that racial/ethnic minorities were less likely than whites to be eligible for dysglycemia screening. Similarly, one population-based study found that screening eligibility based on age ≥45 y was lower among Blacks and Hispanics/Latinos compared to whites [42]. Nonwhites in our study had a higher risk of developing dysglycemia than whites, a finding that is well established in the epidemiologic literature. We found that dysglycemia develops at younger ages and lower body weight in racial/ethnic minority groups, which is supported by a recent body of research [16–18]. In addition, the proportion of incident dysglycemia cases detected by the USPSTF criteria was lower among all racial/ethnic groups than among whites. Taken together, these observations raise concern that the current USPSTF screening criteria may postpone a diagnosis of dysglycemia in racial/ethnic minorities, which could potentially delay interventions to prevent or treat diabetes in these groups. Prospective studies in which all individuals receive screening are needed to further investigate the performance of these criteria and to assess whether following them contributes to diabetes-related disparities [43]. In contrast to the current USPSTF screening criteria, other diabetes screening guidelines include nonwhite race/ethnicity as a risk factor that warrants screening independent of age or weight [22,44,45].

Because prediabetes represented the majority of incident dysglycemia cases, our findings have implications for diabetes prevention among disadvantaged US adults. The low sensitivity of current USPSTF screening criteria reported here could result in missed opportunities to offer intensive lifestyle interventions or metformin. Adults with prediabetes who are missed by these screening criteria will also not learn that they have the condition, which may impact their efforts to prevent diabetes. One recent study found that adults who were aware of having prediabetes were more than twice as likely as those who were not to engage in meeting evidence-based lifestyle goals [46]. Without receiving any intervention to lower their diabetes risk, patients with prediabetes who are not detected by the USPSTF screening criteria may develop diabetes sooner and have a longer period of exposure for developing complications. Future research should investigate the impact of screening positive for prediabetes on the adoption of diabetes prevention treatments and resulting metabolic outcomes.

Strengths of the current study include its timeliness given the recent release of the 2015 USPSTF screening recommendation, large sample size, and use of longitudinal clinical data from an understudied population in the US. The sociodemographic characteristics of our cohort were similar to those reported among over 15 million adult patients served in similar community health centers [19]. While these community health centers are the primary source of outpatient care for immigrants living in the US [47]—many of whom are racial/ethnic minorities—the performance of these diabetes screening criteria may differ in their countries of origin. However, diabetes also develops at young ages and lean body weight among adults in Latin America, Africa, and Asia [48–52]. This suggests that screening programs targeting middle-aged and overweight/obese adults may also have low sensitivity for detecting dysglycemia in these regions [53].

Our analysis has the following limitations. We did not have individual-level data for patients who were excluded because they had fewer than two follow-up visits, which precluded analysis of differences between the study sample and the larger patient population from which it was drawn. We were only able to examine the effectiveness of current USPSTF screening criteria among patients who had available test results or evidence of a new diagnosis code or treatment, which were required to ascertain whether patients developed dysglycemia during follow-up. Therefore, the performance characteristics reported here (i.e., sensitivity, specificity, PPV, and NPV) may differ from those in the entire patient sample because of unobserved factors associated with being screened. However, the findings from this sample of tested patients are relevant to practicing clinicians, who similarly lack data on glycemic status among patients without available test results. In addition, one main contribution of our study is identifying patient characteristics associated with screening eligibility, which were derived from the entire study sample. These findings suggest how the criteria may perform if implemented with fidelity in a similar patient population. Because of differences between the current sample and patients who receive primary care in other settings, our findings may have limited generalizability outside of safety-net clinics like the ones studied here.

The ascertainment of dysglycemia was based on a single glycemic test result. The intraindividual variation observed with these tests may have resulted in misclassification of cases. However, this limitation did not likely have a large impact on our findings because the two tests with the least variability (A1C [coefficient of variation <1%] and fasting glucose [coefficient of variation 5.7%]) were used to ascertain the outcome in 96.1% of cases [54]. Two-hour postload glucose, which has the greatest intraindividual variability (coefficient of variation 16.7%), was used to define dysglycemia in only 0.7% of cases [54]. Because the current USPSTF criteria were not recommended during the 2008–2013 study period, we could not investigate how this guideline influenced providers’ screening behavior.

### Conclusions and Implications

Our findings suggest that the 2015 USPSTF screening recommendation may identify approximately half of US community health center patients with undiagnosed prediabetes and diabetes. Future studies are needed to estimate the performance of these screening criteria in population-based samples in which all individuals are screened. EHR data can enable pragmatic identification of patients who are eligible for diabetes screening or evidence-based interventions to prevent or treat diabetes. For those who have undiagnosed dysglycemia and are not detected by the USPSTF screening criteria, such interventions may be delayed. Also concerning is the fact that certain high-risk groups who develop dysglycemia at younger ages and lower body weight may be missed by following this screening recommendation, most notably racial/ethnic minorities. Our findings suggest that primary care providers should consider screening patients from racial/ethnic minority groups before the age and weight ranges recommended by the USPSTF. Such a practice could enable earlier management of dysglycemia in these high-risk groups but may impose cost sharing on patients.

## Supporting Information

S1 STROBE Checklist STROBE Checklist. (RTF)

## Abbreviations

A1C: hemoglobin A1C
ADA: American Diabetes Association
Alliance: Alliance of Chicago Community Health Services
BMI: body mass index
CHARN: Community Health Applied Research Network
CI: confidence interval
EHR: electronic health record
ICD-9: International Classification of Diseases, Ninth Revision
NPV: negative predictive value
PPV: positive predictive value
USPSTF: United States Preventive Services Task Force
