# Supplementary material for: Detecting Dysglycemia Using the 2015 United States Preventive Services Task Force Screening Criteria: A Cohort Analysis of Community Health Center Patients
Source: PLoS Med. 2016 Jul 12;13(7):e1002074. doi: 10.1371/journal.pmed.1002074 (PMC4942097; doi:10.1371/journal.pmed.1002074)
Supplement: S1 STROBE Checklist — (RTF) [file pmed.1002074.s001.rtf]

STROBE Statement—checklist of items that should be included in reports of observational studies


	Item 
No	
Recommendation	
Explanation and Elaboration		
Title and abstract	1	(a) Indicate the study's design with a commonly used term in the title or the abstract	Detecting Dysglycemia in a Cohort of Minority-Predominant Community Health Center Patients: 2015 U.S. Preventive Services Task Force Screening Criteria		
		(b) Provide in the abstract an informative and balanced summary of what was done and what was found	Please see abstract		
Introduction					
Background/rationale	2	Explain the scientific background and rationale for the investigation being reported	In October 2015, [the USPSTF] issued a new recommendation to screen asymptomatic adults for dysglycemia who are 40 to 70 years old and overweight or obese using one of the following tests: hemoglobin A1C (A1C), fasting plasma glucose, or 2-hour postload glucose during a 75g oral glucose tolerance test [14]… Recent research demonstrating a higher prevalence of diabetes at younger ages and lower body weight [15], particularly among racial/ethnic minorities [16-18], suggests that the USPSTF criteria may identify proportionately fewer cases of dysglycemia among certain high-risk groups.		
Objectives	3	State specific objectives, including any prespecified hypotheses	The objective of our study is to evaluate the performance of the USPSTF screening criteria in detecting dysglycemia among U.S. community health center patients. We hypothesized that racial/ethnic minorities would be less likely to be detected by these screening criteria, yet more likely to develop dysglycemia over time.		
Methods					
Study design	4	Present key elements of study design early in the paper	In this retrospective cohort study, we analyzed longitudinal electronic health record (EHR) data from a U.S. network of publicly-funded community health centers between 2008 and 2013.		
Setting	5	Describe the setting, locations, and relevant dates, including periods of recruitment, exposure, follow-up, and data collection	EHR data from routine primary care encounters were collected retrospectively by Alliance of Chicago Community Health Services (Alliance), a Health Center Controlled Network and member of the Community Health Applied Research Network (CHARN). The affiliated safety-clinics offer comprehensive clinical services to vulnerable populations including large proportions of women and racial/ethnic minorities [19]. Six community health centers in the CHARN network serving patients in the American Midwest and Southwest participated in this study [20]… Adult patients at least 18 years old were identified in the EHR between January 2008 and December 2010 based on their first office visit during this time period, defined as the index visit… The final study population included 50,515 patients, who were followed after the index visit for up to three years through December 2013.		
Participants	6	(a) Cohort study—Give the eligibility criteria, and the sources and methods of selection of participants. Describe methods of follow-up
Case-control study—Give the eligibility criteria, and the sources and methods of case ascertainment and control selection. Give the rationale for the choice of cases and controls
Cross-sectional study—Give the eligibility criteria, and the sources and methods of
selection of participants	Adult patients at least 18 years old were identified in the EHR between January 2008 and December 2010 based on their first office visit during this time period, defined as the index visit. Patients who had fewer than two subsequent office visits were excluded after examining all face-to-face encounters with a licensed primary care provider in the participating health centers. We also excluded those with dysglycemia at baseline, determined by the following EHR criteria documented any time before the end of the calendar year in which patients attended the index visit: 1) ICD-9 diagnosis code for prediabetes or diabetes; 2) prescription order for any antidiabetic medication; and/or 3) glycemic testing result consistent with dysglycemia [fasting glucose ≥100mg/dL (5.55mmol/L), 2-hour postload glucose ≥140mg/dL (7.77mmol/L), random glucose ≥200mg/dL (11.10mmol/L), or A1C ≥5.7% (39mmol/mol)… The final study population included 50,515 patients, who were followed after the index visit for up to three years through December 2013.		
		(b) Cohort study—For matched studies, give matching criteria and number of exposed and unexposed
Case-control study—For matched studies, give matching criteria and the number of controls per case	N/A		
Variables	7	Clearly define all outcomes, exposures, predictors, potential confounders, and effect
modifiers. Give diagnostic criteria, if applicable	The development of clinically-detected dysglycemia was the primary outcome… Patients with one of the following glycemic testing results were considered to have dysglycemia: fasting glucose ≥100mg/dL (5.55mmol/L), 2-hour postload glucose ≥140mg/dL (7.77mmol/L), or A1C ≥5.7% (39mmol/mol). Patients with random glucose ≥200mg/dL (11.10mmol/L) were classified as having diabetes and therefore included among those who developed dysglycemia [22]. A label of “fasting,” “random,” or “2-hour postload” for all glucose results in the EHR enabled laboratory definitions of the outcome, which was based on the first result observed during the follow-up period. Dysglycemia was also defined by documentation of diagnosis codes for prediabetes or type 2 diabetes during follow-up. Patients with a new antidiabetic medication prescription were also considered to have dysglycemia. …According to the 2015 USPSTF criteria, we considered all patients aged 40 to 70 years old and overweight or obese to be eligible for screening. In addition to age and body mass index (BMI), we analyzed baseline data on each of the following diabetes risk factors as defined according to the American Diabetes Association (ADA): non-white race/ethnicity, hypertension, dyslipidemia, polycystic ovary syndrome, history of gestational diabetes, and family history of diabetes [22]…We summed the number of diabetes risk factors, including age ≥45 years [22] and those mentioned above, to create an individual risk score. Sociodemographic variables including sex and insurance status were also assessed at the index visit and examined as covariates [23, 24].  
Table 1 presents the diagnostic criteria for all clinical predictors.		
Data sources/ measurement	8*	For each variable of interest, give sources of data and details of methods of assessment (measurement). Describe comparability of assessment methods if there is more than one group	We analyzed longitudinal electronic health record (EHR) data [for all variables].		
Bias	9	Describe any efforts to address potential sources of bias	In a sensitivity analysis, we estimated the performance characteristics of the USPSTF criteria including patients who also had undiagnosed dysglycemia at baseline, who would also be eligible for screening according to the recommendation.		
Study size	10	Explain how the study size was arrived at	Adult patients at least 18 years old were identified in the EHR between January 2008 and December 2010 based on their first office visit during this time period, defined as the index visit. Patients who had fewer than two subsequent office visits were excluded after examining all face-to-face encounters with a licensed primary care provider in the participating health centers. We also excluded those with dysglycemia at baseline, determined by the following EHR criteria documented any time before the end of the calendar year in which patients attended the index visit: 1) ICD-9 diagnosis code for prediabetes or diabetes; 2) prescription order for any antidiabetic medication; and/or 3) glycemic testing result consistent with dysglycemia [fasting glucose ≥100mg/dL (5.55mmol/L), 2-hour postload glucose ≥140mg/dL (7.77mmol/L), random glucose ≥200mg/dL (11.10mmol/L), or A1C ≥5.7% (39mmol/mol)]. These patients were excluded because the study's primary outcome was the development of dysglycemia during a three-year follow-up period. In addition, we excluded five subjects with unknown sex. The final study population included 50,515 patients, who were followed after the index visit for up to three years through December 2013.
Figure 1 clearly displays how the study size  was arrived at.		
Quantitative variables	11	Explain how quantitative variables were handled in the analyses. If applicable, describe which groupings were chosen and why	See copied text above, and also Table 1 in the manuscript.		
Statistical methods	12	(a) Describe all statistical methods, including those used to control for confounding	We used summary statistics to characterize the study cohort with respect to all covariates at baseline. Chi-square tests were used to examine the association between diabetes screening eligibility according to 2015 USPSTF criteria (eligible vs. ineligible) and the following indicators: 1) baseline characteristics, 2) receipt of screening during follow-up; and 3) development of clinically-detected dysglycemia. Using data on dysglycemia incidence and USPSTF eligibility among patients who were screened during follow-up, we assessed the performance characteristics of the USPSTF criteria (sensitivity, specificity, positive predictive value [PPV], and negative predictive value [NPV]) based on available screening test results. Estimation of these performance characteristics did not include those who did not receive a screening test during follow-up, in whom the incidence of dysglycemia was unknown. Also in the screened population, we estimated the odds of developing clinically-detected dysglycemia during follow-up using logistic regression adjusted for all the covariates, in addition to community health center site.		
		(b) Describe any methods used to examine subgroups and interactions	We conducted stratified analyses by race/ethnicity to examine the development of dysglycemia separately in these groups.		
		(c) Explain how missing data were addressed	Participants with missing race/ethnicity data were included in the bivariate and multivariable analyses as part of the Other category. Those with missing data for insurance status were included in all analyses under the Missing category. 		
		(d) Cohort study—If applicable, explain how loss to follow-up was addressed
Case-control study—If applicable, explain how matching of cases and controls was addressed
Cross-sectional study—If applicable, describe analytical methods taking account of sampling strategy	N/A		
		(e) Describe any sensitivity analyses	In a sensitivity analysis, we estimated the performance characteristics of the USPSTF criteria including patients who also had undiagnosed dysglycemia at baseline, who would also be eligible for screening according to the recommendation.		
Results					
	13*	(a) Report numbers of individuals at each stage of study—eg numbers potentially eligible, examined for eligibility, confirmed eligible, included in the study, completing follow-up, and analysed	The Figure 1 flow diagram displays the numbers of the total patient population (n=112,662), the patients excluded and reasons for their exclusion (n=62,147), the participants comprising the study sample (n=50,515), and those analyzed (n=50,515). 		
		(b) Give reasons for non-participation at each stage	See Figure 1 and Methods for exclusion criteria.		
		c) Consider use of a flow diagram	See Figure 1		
Descriptive data	14*	(a) Give characteristics of study participants (eg demographic, clinical, social) and information on exposures and potential confounders	At baseline, the patient cohort was predominantly less than 40 years old (62.7%), overweight/obese (66.4%), non-white (77.3%), women (72.5%), and uninsured or publicly insured (74.3%). Over 95% of the sample had at least one risk factor for diabetes, and 25.1% met current USPSTF criteria for diabetes screening. According to these criteria, all patients less than 40 years old or with a normal body weight would have been ineligible for screening. In addition, the following patient characteristics were significantly associated with being ineligible: non-white race/ethnicity, female sex, history of gestational diabetes, and polycystic ovary syndrome (Table 2).		
		(b) Indicate number of participants with missing data for each variable of interest	There were missing data for race/ethnicity (n=977, 1.9%) and insurance status (n=4,103, 8.1%), but not for other variables.		
		(c) Cohort study—Summarise follow-up time	The mean follow-up time was 1.9 years and the maximum was 2.9 years.		
Outcome data	15*	Cohort study—Report numbers of outcome events or summary measures over time	Overall, 29,946 patients (59.3% of the total sample) underwent a screening test within three years of the index visit…Based on available test results, a total of 8,478 patients had incident dysglycemia during the follow-up period [5,960 with prediabetes (70.3% of dysglycemia cases) and 2,518 with diabetes].		
		Case-control study—Report numbers in each exposure category, or summary measures of exposure	N/A		
		Cross-sectional study—Report numbers of outcome events or summary measures	N/A		
Main results	16	(a) Give unadjusted estimates and, if applicable, confounder-adjusted estimates and their precision (eg, 95% confidence interval). Make clear which confounders were adjusted for and why they were included	…the 2015 USPSTF criteria had the following test performance characteristics (95% CI) for identifying dysglycemia: sensitivity 45.0% (43.9%-46.1%), specificity 71.9% (71.3%-72.5%), PPV 38.8% (37.8%-39.7%), and NPV 76.8% (76.2%-77.4%)… The multivariable model of incident dysglycemia adjusted for all potentially confounding variables, including community health center site. The following demographic and clinical characteristics were significantly associated with developing dysglycemia during follow-up: age ≥40 years, overweight and obesity, non-white race/ethnicity, hypertension, polycystic ovary syndrome, history of gestational diabetes, and family history of diabetes (Table 4). The odds of developing dysglycemia increased with greater numbers of diabetes risk factors (Table 4). [ORs and 95% CI for incident dysglycemia are presented in Table 4]		
		(b) Report category boundaries when continuous variables were categorized	N/A		
		c) If relevant, consider translating estimates of relative risk into absolute risk for a meaningful time period	N/A		
Other analyses	17	Report other analyses done—eg analyses of subgroups and interactions, and sensitivity analyses	Racial/ethnic minorities were significantly less likely to be eligible for screening based on the USPSTF recommendation than were whites, with the greatest disparity occurring among Hispanics/Latinos (Table 2). A higher proportion of racial/ethnic minorities who were screened developed clinically-detected dysglycemia during the follow-up period (Figure 1). This was supported by multivariable logistic regression analysis showing higher odds of dysglycemia among all racial/ethnic minority groups compared to whites [odds ratio (95% confidence interval) for Blacks 1.24 (95% CI, 1.09-1.40); Hispanics 1.46 (95% CI, 1.30-1.64); and Other 1.33 (95% CI, 1.16-1.54)] (Table 4). In addition, dysglycemia cases among minority patients were less likely to be identified by the USPSTF screening criteria (i.e. lower sensitivity) than were cases among whites. The sensitivity of the criteria was 54.5% in whites, 50.3% in Blacks, 42.0% in Other, and 37.7% in Hispanic/Latinos (Figure 1). The lower sensitivity observed among racial/ethnic minorities reflects the greater proportion of patients who developed dysglycemia at a normal weight and ages less than 40 within these groups, relative to whites. (Table 5)…
A sensitivity analysis including individuals who had undiagnosed dysglycemia at baseline did not substantively impact the reported USPSTF performance characteristics.		
Discussion					
Key results	18	Summarise key results with reference to study objectives	Using longitudinal data from U.S. community health center patients, we report eligibility for diabetes screening according to the 2015 USPSTF criteria, as well as receipt of screening and positive test results within a three-year follow-up period. Overall, one-quarter of patients were eligible for screening and almost 60% received a screening test during follow-up. Less than half of patients who developed dysglycemia would have been eligible for screening according to current USPSTF criteria. Our findings also suggest that certain population groups that are at high risk for developing dysglycemia are more likely to be missed when using these criteria, particularly racial/ethnic minorities.
		
Limitations	19	Discuss limitations of the study, taking into account sources of potential bias or imprecision.
Discuss both direction and magnitude of any potential bias	Our analysis has the following limitations. We did not have individual-level data on patients who were excluded because they had fewer than two follow-up visits, which precluded analysis of differences between the study sample and the larger patient population from which it was drawn. We were only able to examine the effectiveness of current USPSTF screening criteria among patients who had available test results or evidence of a new diagnosis code or treatment, which were required to ascertain whether patients developed dysglycemia during follow-up. Therefore, the performance characteristics reported here (i.e. sensitivity, specificity, PPV, and NPV) may differ from those in the entire patient sample due to unobserved factors associated with being screened. However, the findings from this sample of tested patients are relevant to practicing clinicians, who similarly lack data on glycemic status among patients without available test results. In addition, one main contribution of our study is identifying patient characteristics associated with screening eligibility, which were derived from the entire study sample. These findings suggest how the criteria may perform if implemented with fidelity in a similar patient population…
The ascertainment of dysglycemia was based on a single glycemic test result. The intraindividual variation observed with these tests may have resulted in misclassification of cases. However, this limitation did not likely have a large impact on our findings because the two tests with the least variability (A1C and fasting glucose) were used to ascertain the outcome in 96.1% of patients. Two-hour postload glucose, which has the greatest intraindividual variation, was only used to define dysglycemia in only 0.7% of patients. Because the current USPSTF criteria were not recommended during the 2008-2013 study period, we could not investigate how this guideline influenced providers' screening behavior.
		
Interpretation	20	Give a cautious overall interpretation of results considering objectives, limitations, multiplicity of analyses, results from similar studies, and other relevant evidence	Our findings suggest that the 2015 USPSTF screening recommendation may identify approximately half of U.S. community health center patients with undiagnosed prediabetes and diabetes.		
Generalisability	21	Discuss the generalisability (external validity) of the study results	Due to differences between the current sample and patients who receive primary care in other settings, our findings may have limited generalizability outside of safety-net clinics like the ones studied here.  		
Other information					
	22	Give the source of funding and the role of the funders for the present study and, if applicable,
for the original study on which the present article is based	We acknowledge the funders of this study: the National Institute of Diabetes and Digestive and Kidney Diseases, National Institutes of Health (K23DK095981) and the Agency for Healthcare Research and Quality (P01HS21141). The study was also funded in part by a pilot and feasibility grant from the Chicago Center for Diabetes Translation Research (P30DK092949).		

*Give information separately for cases and controls in case-control studies and, if applicable, for exposed and unexposed groups in cohort and cross-sectional studies.
